# Aberrant methylated key genes of methyl group metabolism within the molecular etiology of urothelial carcinogenesis

**DOI:** 10.1038/s41598-018-21932-7

**Published:** 2018-02-22

**Authors:** Lars Erichsen, Foued Ghanjati, Agnes Beermann, Cedric Poyet, Thomas Hermanns, Wolfgang A. Schulz, Hans-Helge Seifert, Peter J. Wild, Lorenz Buser, Alexander Kröning, Stefan Braunstein, Martin Anlauf, Silvia Jankowiak, Mohamed Hassan, Marcelo L. Bendhack, Marcos J. Araúzo-Bravo, Simeon Santourlidis

**Affiliations:** 1 Epigenetics Core Laboratory, Institute of Transplantation Diagnostics and Cell Therapeutics, Medical Faculty, Heinrich-Heine University Duesseldorf, Moorenstr. 5, 40225 Duesseldorf, Germany; 2 Department of Urology, University Hospital, University of Zurich, Zurich, Switzerland; 3 Department of Urology, Medical Faculty, Heinrich-Heine University Duesseldorf, Duesseldorf, Germany; 4 Urologische Klinik, Universitätsspital Basel, Basel, Switzerland; 5 Institute of Surgical Pathology, University Hospital, University of Zurich, 8091 Zurich, Switzerland; 6 Department of Pathology, Medical Faculty, Heinrich-Heine University Duesseldorf, Duesseldorf, Germany; 7 Department of Surgery, Tulane University School of Medicine, New Orleans, LA 70112 USA; 8 Institut National de la Santé et de la Recherché Médicale, University of Strasbourg, 67000 Strasbourg, France; 9 Department of Urology, University Hospital, Positivo University, Curitiba, Brazil; 10 Group of Computational Biology and Systems Biomedicine, Biodonostia Health Research Institute, 20014 San Sebastián, Spain; 11 IKERBASQUE, Basque Foundation for Science, 48009 Bilbao, Spain

## Abstract

Urothelial carcinoma (UC), the most common cancer of the urinary bladder causes severe morbidity and mortality, e.g. about 40.000 deaths in the EU annually, and incurs considerable costs for the health system due to the need for prolonged treatments and long-term monitoring. Extensive aberrant  DNA methylation is described to prevail in urothelial carcinoma and is thought to contribute to genetic instability, altered gene expression and tumor progression. However, it is unknown how this epigenetic alteration arises during carcinogenesis. Intact methyl group metabolism is required to ensure maintenance of cell-type specific methylomes and thereby genetic integrity and proper cellular function. Here, using two independent techniques for detecting DNA methylation, we observed DNA hypermethylation of the 5′-regulatory regions of the key methyl group metabolism genes *ODC1*, *AHCY* and *MTHFR* in early urothelial carcinoma. These hypermethylation events are associated with genome-wide DNA hypomethylation which is commonly associated with genetic instability. We therefore infer that hypermethylation of methyl group metabolism genes acts in a feed-forward cycle to promote additional DNA methylation changes and suggest a new hypothesis on the molecular etiology of urothelial carcinoma.

## Introduction

Urothelial carcinoma (UC), the most common cancer of the urinary bladder, causes severe morbidity and substantial mortality. Each year, approximately 110 500 men and 70 000 women are diagnosed with the disease and 38 200 patients in the European Union and 17 000 patients in the US die from UC^1^. Approximately 75% of bladder cancer is non-muscle invasive (NMI) UC at the time of diagnosis, of which 70% present as noninvasive, papillary tumors confined to the mucosa (pTa), 20% as tumors invading the subepithelial tissue (pT1), and 10% as flat dysplastic (carcinoma *in situ*, or CIS) lesions^2^. High grade (HG) tumors carry a worse prognosis than low-grade (LG) tumors. Relapse rates typically range from 30% to 70% with rates of progression as high as 10–30% for high-grade tumors^3^. Furthermore, due to this high tendency towards relapsing, UC places an enormous burden on health care systems^3^. About one-third of all bladder cancers occur as multifocal disease with several tumors forming at different sites of the bladder wall^4^. It is thought that a “field defect” underlies both high relapse rates and multifocality. According to this hypothesis, urothelial cells in regions adjacent to the tumors are already primed to undergo transformation because their genetic integrity has been already disturbed by environmental mutagens^4^.

DNA methylation is a fundamental epigenetic mechanism, involved in organization of the genome and mediating effects of environmental influences on adaptive regulation of gene expression^5,6^. Exposure to harmful environmental stimuli can affect proper epigenetic gene regulation and as a consequence can lead to impairment of cellular differentiation and functions.

In urothelial carcinoma, focal DNA hypermethylation events, often associated with gene repression, and genome-wide LINE-1 hypomethylation have been detected in non-muscle invasive as well as in more aggressive muscle-invasive tumors^7,8^. LINE-1 hypomethylation is present in up to 90% of UC^9^ and has been pursued as a potential potent biomarker for early diagnosis of UC and monitoring of recurrent disease. It may contribute to carcinogenesis in various ways, including induction of genomic instability^10^ and aberrant transcription patterns^11^.

Exposure to various chemical carcinogens such as occupational exposure to aromatic amines, and polycyclic aromatic hydrocarbons, with different attributable risks, is recognized as an important risk factor in urothelial carcinogenesis^1^. In addition, exposure to arsenic in drinking water and aristolochic acid in food or remedies, have been recognized as further causes of UC. The single main risk factor for bladder cancer is tobacco smoking^12^, which is estimated to account for 50% of tumors^13^. Tobacco smoke contains aromatic amines, such as 2-naphthylamine, and polycyclic aromatic hydrocarbons that are renally excreted and exert their carcinogenic effect on the entire urinary tract. They are thought to act as carcinogens by forming DNA adducts and causing mutations in key cancer-related genes^14^. Consequently quitting smoking reduces bladder cancer risk. Furthermore, higher fluid intake may reduce exposure of urothelial tissue to these carcinogens by diluting urine and increasing the frequency of micturition^1^. Indeed, Michaud *et al*. presented a case-control study finding water intake to be inversely associated with bladder UC risk^15^.

Accumulating evidence indicates that aberrant DNA methylation occurs as a direct consequence of exposure of urothelial cells to tobacco smoke carcinogens in urine^16^. For instance, in an *in vitro* model tobacco smoke induced the stepwise transformation of urinary tract epithelial cells with concomitant hypermethylation of many genes^17^. Similarly, several studies have linked arsenic exposure with hypermethylation and hypomethylation events in urothelial carcinogenesis (see refs in Schulz & Goering^8^).

Consequently it has been suggested that one main goal of an urologist should be to inform smoking patients on the causative factors of UC and to strongly counsel to stop smoking^18^.

Despite these insights, the mechanisms leading to the progressive changes in DNA methylation during urothelial carcinogenesis are still largely unclear. We were therefore intrigued to observe during a screen for DNA methylation alterations in early stage urothelial carcinoma and adjacent, morphologically normal urothelium that genes encoding crucial enzymes in methyl group metabolism pathways appeared themselves to be affected by aberrant DNA methylation. To follow up on this observation, we applied two independent techniques for the detection of DNA methylation. The results indeed provide evidence that key genes of methyl group metabolism are themselves affected by aberrant DNA methylation in their 5′-regulatory regions in early stage urothelial carcinoma. These findings implicate a new mechanism in the etiology of epigenetic alterations during UC carcinogenesis.

## Results

### Detection of differential methylated key genes of methylgroup metabolism by global DNA methylation array analyses in UC

There is evidence that tumor adjacent uroepithelium, although it might pathologically be reviewed to consist of healthy uroepithelial cells, is already in a premalignant state which is characterized by genetic, e.g. TP53 mutant cells^4^, and widespread epigenetic alterations, e.g. hypermethylated genes which are also present in bladder cancer cells^19^. It has been suggested that this “field defect” predispose the epithelial tissue to undergo transformation and might underlie tumor recurrence and multifocality, both hallmarks of bladder cancer. Genome-wide DNA methylation data sets were generated from pathologically classified UC, tumor-adjacent normal-appearing and healthy urothelial tissue samples by MeDIP and promoter array analyses (see methods). The reference group, to which all other groups were compared, consisted of 4 tissue samples of healthy urothelium. The other 4 groups of tissue samples respectively consisted of 10 specimens of UC from unifocal papillary tumors (UT), 4 specimens adjacent to unifocal tumors, histologically verified as normal urothelium (badj UT), 5 tissue specimens of UC from multifocal tumors (MT) and 5 specimens adjacent to multifocal tumors, histologically verified as normal urothelium (badjMT) (Table 1).

**Table 1:** Overview of used healthy and tumor-adjacent urothelial and UC tissue samples.

Sample ID	Stage/Grade	Localisation	Sample origin
35396	Healthy bladder tissue	—	microdissection
1229–4	Healthy bladder tissue	—	microdissection
AC1	Healthy bladder tissue	—	punch
AC3	Healthy bladder tissue	—	punch
30833	pTa LG	unifocal	microdissection
28923	pTa LG	unifocal	punch
108T	pTa LG	unifocal	punch
105T	pTa HG	unifocal	punch
111T	pTa HG	unifocal	punch
28643	pT1 LG	unifocal	punch
29986	pT1 HG	unifocal	punch
00156	pT1 HG	unifocal	punch
151T	pT1 HG	unifocal	punch
28774	pT3 HG	unifocal	microdissection
108NT	pTa LG adjacent	unifocal	punch
105NT	pTa HG adjacent	unifocal	punch
111NT	pTa HG adjacent	unifocal	punch
151NT	pT1 HG adjacent	unifocal	punch
51T	pTa LG	multifocal	punch
148T	pTa LG	multifocal	punch
84T	pTa HG	multifocal	punch
63T	pT1 HG	multifocal	punch
103T	pT1 HG	multifocal	punch
51NT	pTa LG adjacent	multifocal	punch
148NT	pTa LG adjacent	multifocal	punch
84NT	pTa HG adjacent	multifocal	punch
63NT	pT1 HG adjacent	multifocal	punch
103NT	pT1 HG adjacent	multifocal	punch

**Figure 1:**
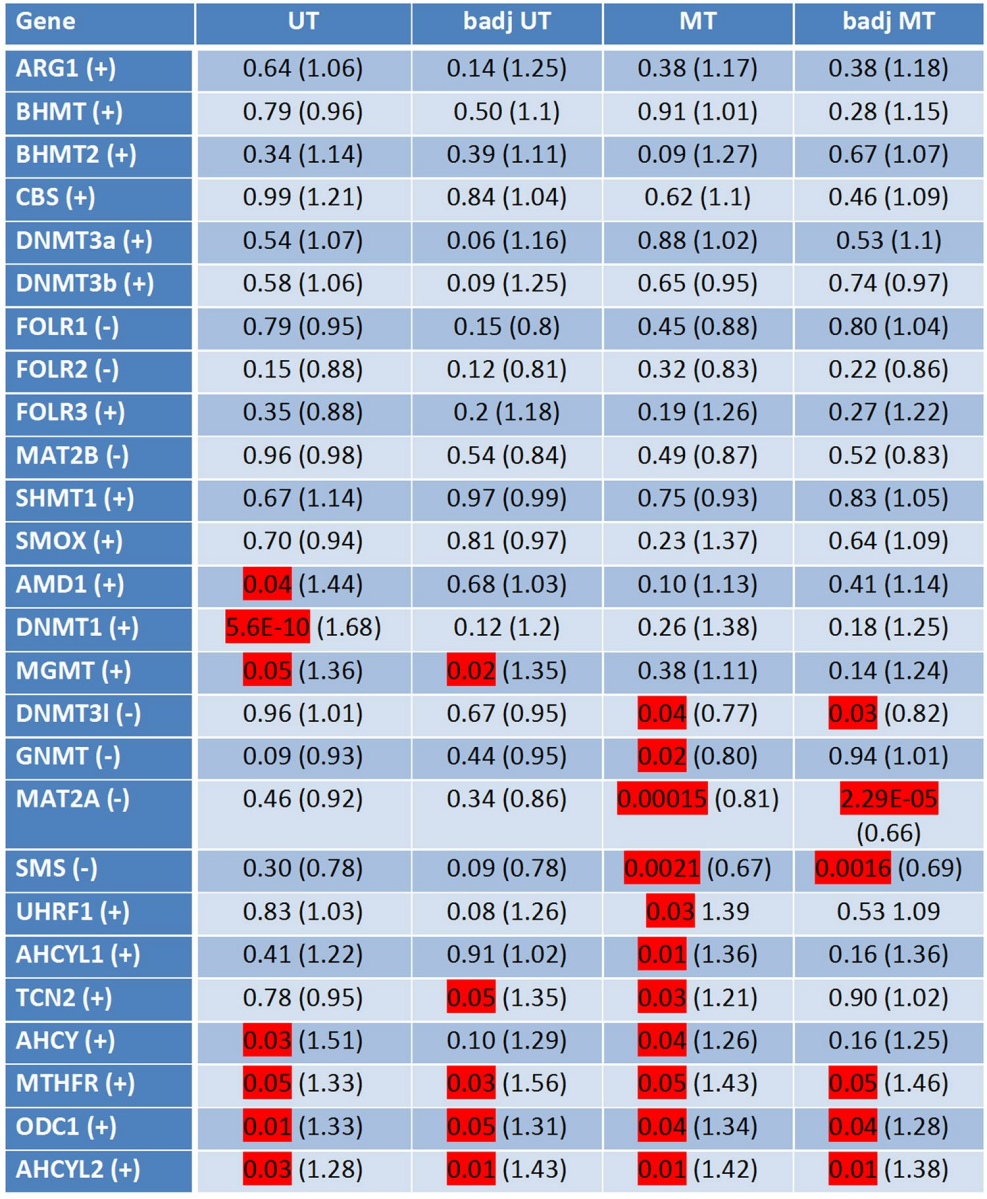
Differences in methylation in 5′ regulatory regions of key methyl group metabolism genes in urothelial carcinoma. Differentially methylated regions (DMRs) were detected by MeDIP/promoter array analyses applied on 4 reference specimens of healthy urothelium, compared to 10 UC tissue specimens from unifocal tumors (UT), 4 specimens adjacent to a unifocal tumor, histologically verified as benign, normal urothelial tissue (badj UT), 5 UC tissue specimens from multifocal tumors (MT) and 5 specimens adjacent to a multifocal tumor, histologically verified as benign, normal urothelial tissue (badj MT). Statistical significance of the detected differential methylation in the tumors and tumor adjacent tissue groups was evaluated using the two sample Student’s t-test. The table lists p-values and fold-changes in parentheses. Values of significant occurrence are highlighted in red. “+” stands for hypermethylation and “−” represents hypomethylation of the respective gene’s DMR.

For each individual sample, integrated peak values for overall methylation of CpG-islands associated with the 5′-regions of methyl group metabolism genes were obtained. Mean values of these peak values were calculated for each sample group. Supplementary Fig. 1 exemplarily illustrates the detailed differences of mean peak values of 4 differentially methylated genes of each group. The statistical significance of differentially methylated regions (DMRs) was calculated for each group of samples compared to the healthy urothelium reference, based on the mean peak values by two-sample Student’s t-test with a significance threshold α_DMR_ = 0.05. The results are summarized in Fig. 1 and provide a first overview of these differentially methylated genes (Fig. 1). They can be divided into 3 main groups. Twelve of 26 considered genes did not show statistically significant differential methylation in any of the sample groups compared to healthy urothelium, despite a trend towards hypermethylation in *ARG1, BHMT, BHMT2, CBS, DNMT3A, DNMT3B, FOLR3, SHMT1 and SMOX*, and a trend towards hypomethylation in *FOLR1/2* and *MAT2B* and in the corresponding tumor-adjacent, histologically benign urothelium.

The second group consisted of genes with statistically significant hypermethylation in either unifocal or multifocal tumors, namely *AMD1, DNMT1 and MGMT* in unifocal tumors and *UHRF1, AHCYL1 and TCN2* in multifocal tumors. Noteworthy, *MGMT* was also significantly hypermethylated in histologically benign urothelial tissue samples adjacent to unifocal tumors. *GNMT*, *DNMT3l, MAT2A*, and *SMS* were hypomethylated in multifocal tumors, and the last three of these genes were also hypomethylated in the multifocal tumors adjacent, histologically benign urothelial tissue samples.

Finally *ODC1, AHCY, MTHFR* and *AHCYL2* were significantly hypermethylated in their 5′-regulatory gene regions in unifocal as well as multifocal tumors. Significant hypermethylation of *ODC1, MTHFR* and *AHCYL2* occured also in the tumor adjacent, histologically benign urothelial tissue samples (Fig. 1). The differential methylation of these genes in bladder cancer was not apparent in previously published data by other groups (see refs^19–22^). However, a direct comparison with The Cancer Genome Atlas (TCGA) expression dataset on the other hand revealed significant lower expression of the hypermethylated genes *MTHFR* and *ODC1* but not of *AHCY* in bladder cancer samples (Suppl. Figure 3).

### Verification of DNA methylation changes of *ODC1* and *AHCY in UC* by bisulfite sequencing

**Figure 2:**
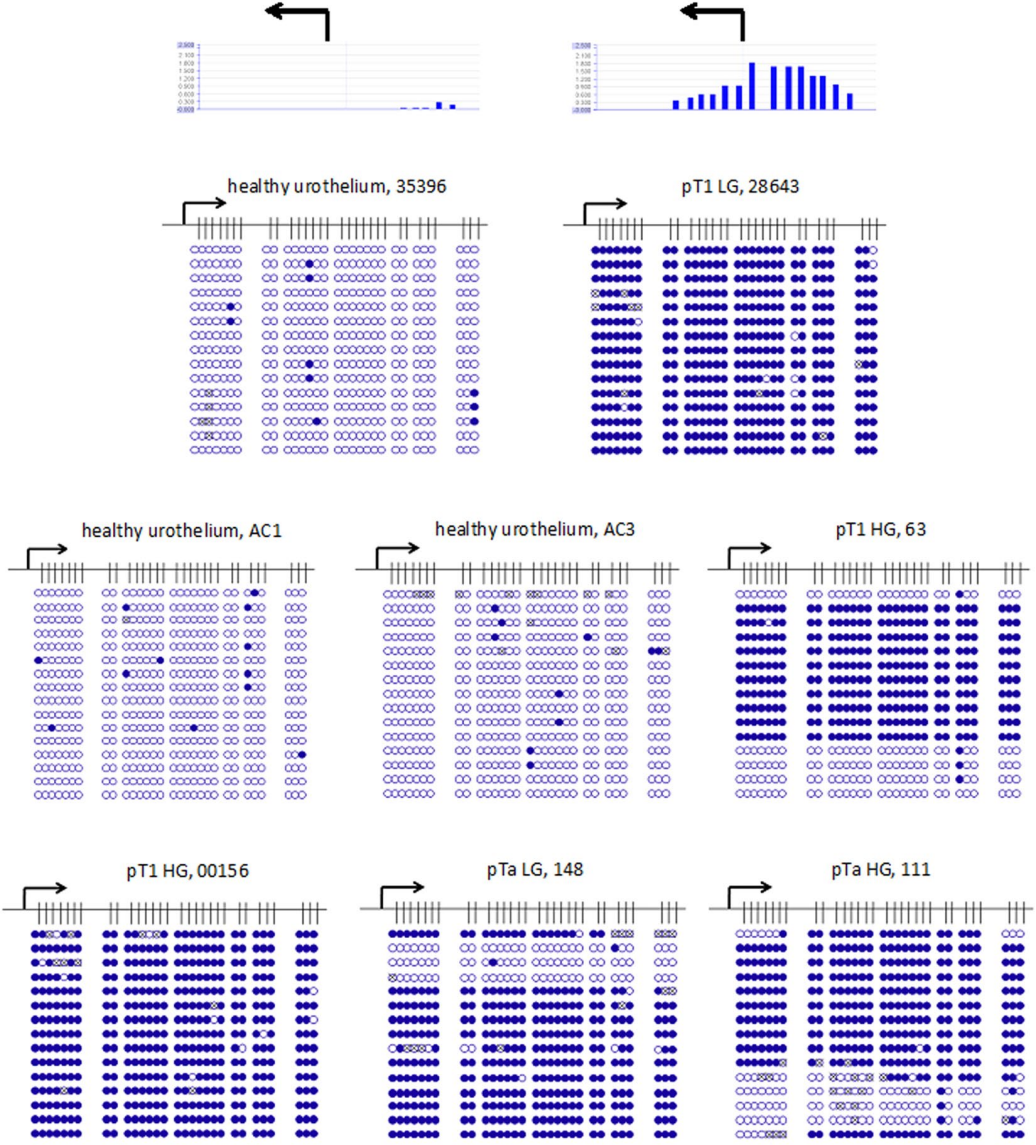
Detailed analyses by bisulfite sequencing of CpG island methylation patterns within 5′ regulatory region of *ODC1* gene in early urothelium carcinoma. Stage and grade of UC tissue specimens with sample IDs (Table 1) are depicted above the panels. Exemplified above the first two panels, two corresponding array results are shown by bar plots. Each blue bar represents a 75 bp long CpG rich probe on the array. The high of a bar is proportional to methylation degree. Below the detailed CpG methylation profiles of the *ODC1* 5′-regulatory region are documented as revealed by bisulfite sequencing. Filled circles stand for methylated CpG dinucleotides. White cyrcles stand for unmethylated CpGs. Crossed cyrcles stand for undefined CpG methylation status. Arrows indicate the transcription start site of *ODC1* gene.

In order to confirm the hypermethylation detected in *ODC1* and *AHCY* by MeDIP-array analyses and to reveal the detailed methylation patterns of these differentially methylated regions (DMRs) we used standard bisulfite sequencing. Specifically, *ODC1* hypermethylation had been detected in two pTaLG (2xMT), in two pTaLGadj (1xMT, 1xUT), in three pTaHG (1xMT, 2xUT), in two pTaHGadj (2xUT), in four pT1HG (2xMT, 2xUT), and in three pT1HGadj (1xMT, 2xUT) tissue samples. Exemplarily, bisulfite sequencing confirmed complete lack of DNA methylation of the CpG-rich *ODC1* 5′-region in three DNA samples from healthy urothelium (Fig. 2). Conversely, dense methylation of the same region was exemplarily confirmed for one pTa low grade and one pTa high grade UC samples (Fig. 2). In addition, dense methylation was also confirmed in one low and two high grade primary pT1 UC tissue samples (Fig. 2). In all these cases DNA methylation array data had indicated DNA hypermethylation of the respective *ODC1* 5′-regulatory region. Noteworthy, two pT1 samples identified as not hypermethylated in the array analysis were also completely unmethylated according to bisulfite sequencing (data not shown). Thus, hypermethylation at the 5′-region of *ODC1* appears to occur in some, but not all early stage urothelial carcinoma specimens.

**Figure 3:**
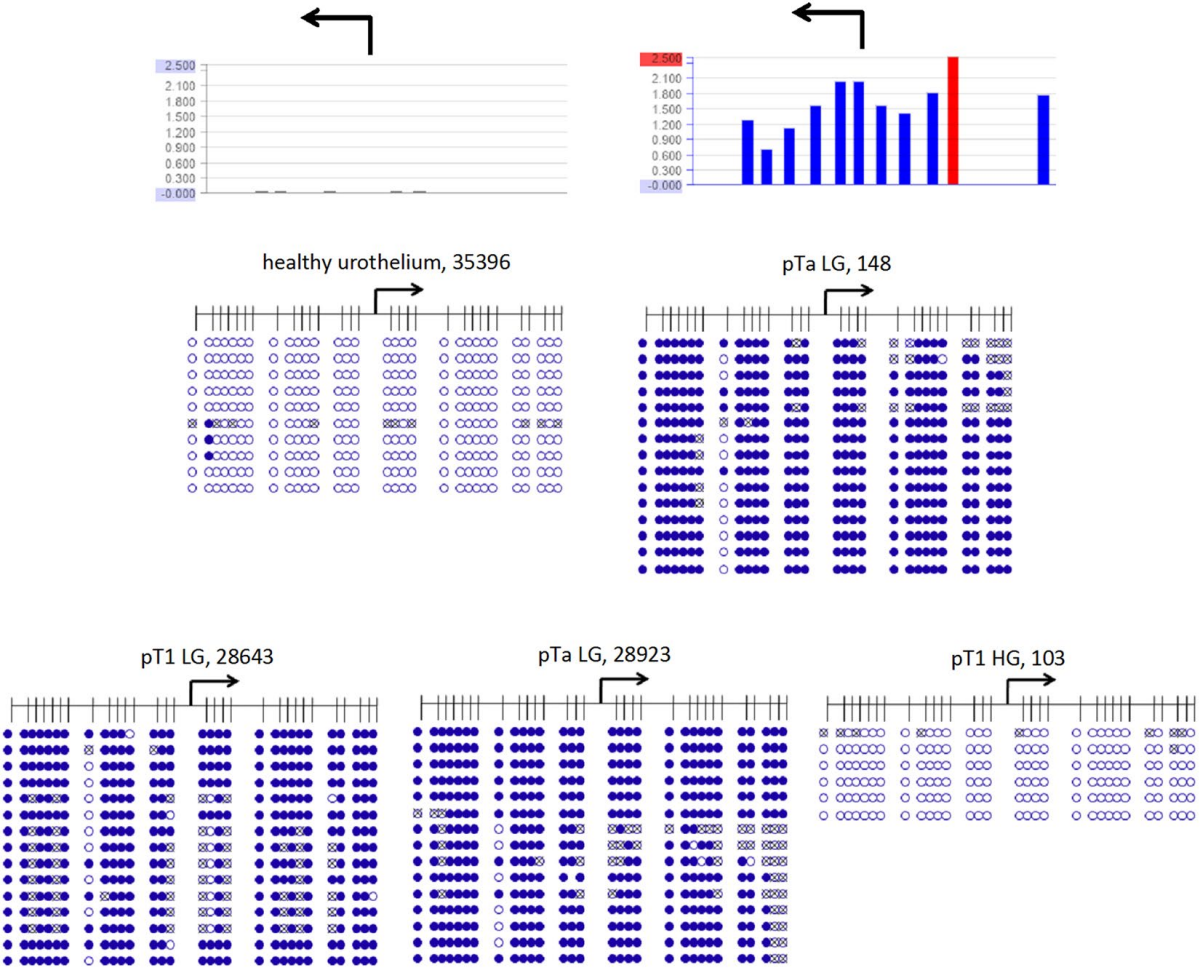
Detailed analysis by bisulfite sequencing of CpG island methylation patterns within 5′ regulatory region of *AHCY* gene in early urothelium carcinoma. Stage and grade of UC tissue specimens with sample IDs (Table 1) are depicted above the panels. Exemplified above the first two panels, two corresponding array results are shown by bar plots. Each blue bar represents a 75bp long CpG rich probe on the array. The high of a bar is proportional to methylation degree. Underneath the detailed CpG methylation profiles of the CpG rich 5′ regulatory region of *AHCY* gene are documented as they were revealed by bisulfite sequencing. Filled circles stand for methylated CpG dinucleotides. White circles stand for unmethylated CpG. Crossed out circles stand for undefined CpG methylation status. Arrows indicate the transcription start site of *AHCY* gene. The red bar within the above, right bar diagram indicates that the corresponding value is 3200 and thus beyond the scale.

According to the array analysis, the CpG-island across the *AHCY* 5′-regulatory region was completely unmethylated in intact, healthy urothelium samples, whereas *AHCY* hypermethylation was detected in the following tissue samples: 4 pTaLG (2xMT, 2xUT), 1 pTaLGadj (MT), 3 pTaHG (1xMT, 2xUT), 3 pTaHGadj (3xUT), 1 pT1LG (UT), 1 pT1HG (MT), 2pT1HGadj (1xMT, 1xUT). We confirmed by bisulfite sequencing dense DNA methylation of this region in one unifocal pTa low grade, one multifocal pTa low grade and one unifocal pT1 low grade UC tissue sample and in contrast, lack of methylation in intact, healthy urothelium (Fig. 3). In addition, we confirmed complete lack of methylation in one multifocal pT1 high grade UC sample in accord with the array data. Thus dense DNA methylation occurs in this gene too, in some early urothelial carcinoma specimens.

**Figure 4:**
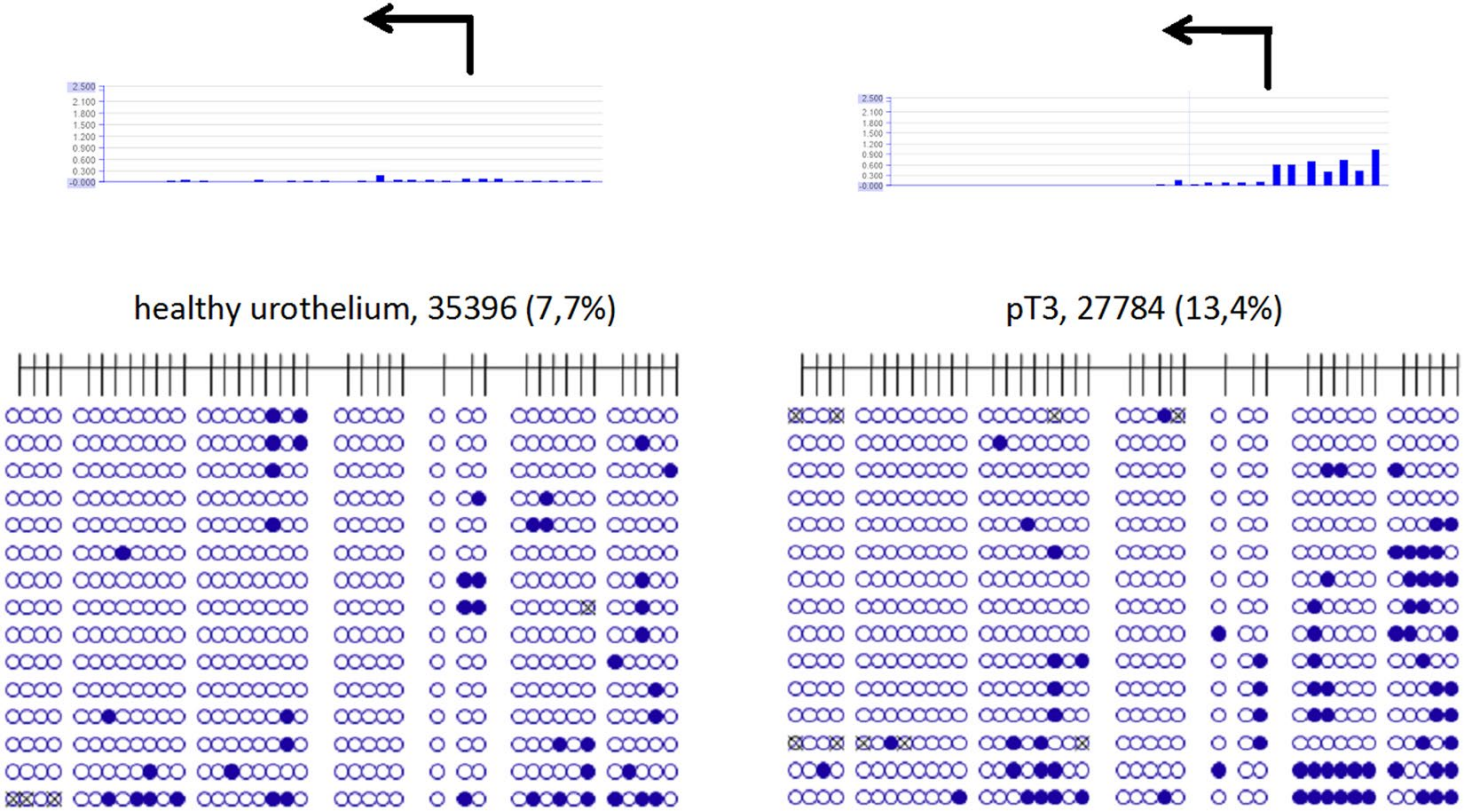
Detailed analysis by bisulfite sequencing of CpG island methylation patterns within 5′- regulatory region of *CBS* gene in urothelium carcinoma. Stage and grade of UC tissue specimens with sample IDs (Table 1) are depicted above the panels. Above the first two panels, two corresponding array results are shown as bar plots. Each blue bar represents a 75 bp long CpG rich probe on the array. The height of a bar is proportional to the methylation degree. Underneath the corresponding detailed CpG methylation profiles of the CpG-rich 5′ regulatory region of CBS *gene* are documented as they were revealed by bisulfite sequencing from healthy urothelium and a pT3 UC tissue sample. Filled circles stand for methylated CpG dinucleotides. White circles stand for unmethylated CpG. Percentages in parentheses are indicating the respective methylated CpG portion. Circles which are crossed out stand for undefined CpG methylation status. Arrows indicate the transcription start site of *CBS* gene.

Whereas the array analysis detected strong differences in the methylation of *ODC1* and *AHCY*, less clear-cut differences were observed in other genes. To validate these changes as well by an independent technique, we deliberately chose one of several samples with slight hypermethylation of the *CBS* gene according to the MeDIP-array analysis for validation by bisulfite sequencing. In this case, too, the marginal methylation increase in a microdissected, unifocal pT3 UC tissue sample was confirmed, whereas a healthy urothelium specimen was unmethylated in accord with the corresponding DNA methylation array data (Fig. 4). Thus, genomic bisulfite sequencing data consistently confirmed the corresponding DNA methylation array data in all investigated cases.

### Unusual focal non-CpG methylation in the 5′-regulatory *MTHFR* region in early UC

**Figure 5:**
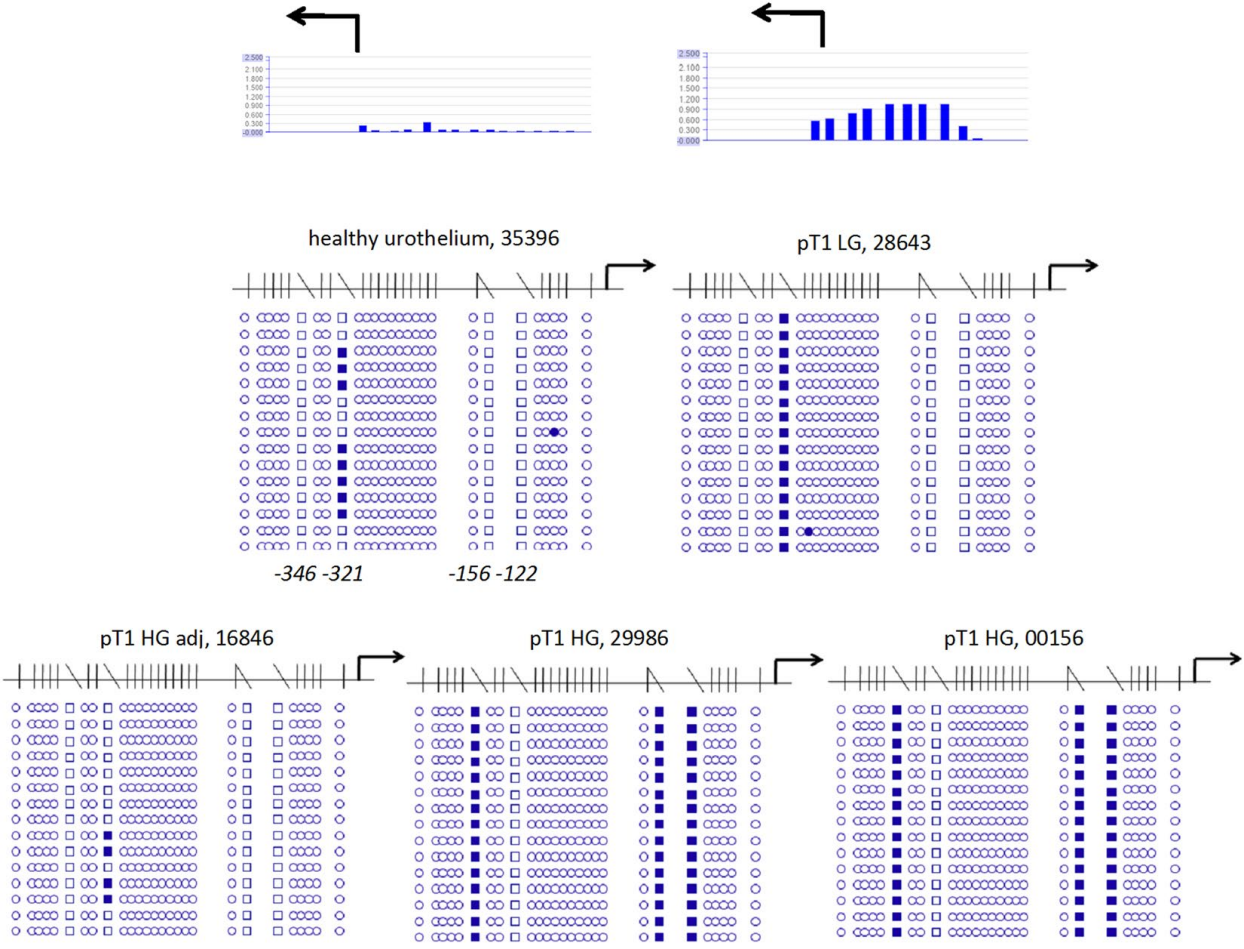
Detailed analysis by bisulfite sequencing of CpG island methylation patterns within 5′ regulatory region of *MTHFR* gene in early urothelium carcinoma. Stage and grade of UC tissue specimens with sample IDs (Table 1) are depicted above the panels. Exemplarily, above the first two panels, two corresponding array results are shown by bar plots. Each blue bar represents a 75 bases long CpG-rich probe on the array. The high of a bar is proportional to the methylation degree. Underneath the detailed CpG methylation profiles of the CpG-rich 5′ regulatory region of MTHFR gene are documented as they were revealed by bisulfite sequencing. Filled rectangles stand for methylated cytosine outside the CpG context. White rectangles stand for unmethylated cytosine. White circles stand for unmethylated CpG dinucleotides. Arrows indicate the transcription start site of *MTHFR* gene. Underneath the upper left panel the positions of the differentially methylated cytosines relative to transcription start site are depicted.

Whereas the reference samples of healthy urothelium lacked methylation according to methylation array analysis, DNA methylation was detected at the *MTHFR* 5′-regulatory region in the following samples: 1 pTaLG (MT), 2pTaLGadj (1xMT, 1xUT), 2 pTaHG (2UT), 2pTaHGadj (2UT), 2 pT1HG (1xMT, 1xUT), 2pT1HGadj (1xMT, 1xUT). Accordingly, bisulfite sequencing revealed increased methylation in one examined unifocal pT1 low grade and in two unifocal pT1 high grade UC tissue specimens compared to one healthy urothelium sample and one pT1 tumor adjacent, benign urothelial tissue specimen (Fig. 5). In the pT1 low grade UC tissue we found all sequences uniformly methylated at cytosine position −321 relative to the transcription start side (NCBI Reference Sequence: NG_013351.1). In both pT1 high grade samples all sequences contained methylated cytosine nucleotides at positions −346, −156 and −122. In the healthy urothelium sample we found only a methylated cytosine at position −321 in a few of the sequences. Thus some early UC samples contained an unusual focal cytosine methylation in the *MTHFR* 5′-regulatory region. Noteworthy, by *in silico* comparison of the differentially methylated nucleotide positions and their sequence contexts with the transcription factor binding sites data bank *Promo*^23^ we found the cytosines −156 and −122 to be located within a predicted glucocorticoid receptor binding site with a dissimilarity margin less than 9%.

### LINE-1 hypomethylation *in* early stage UC

**Figure 6:**
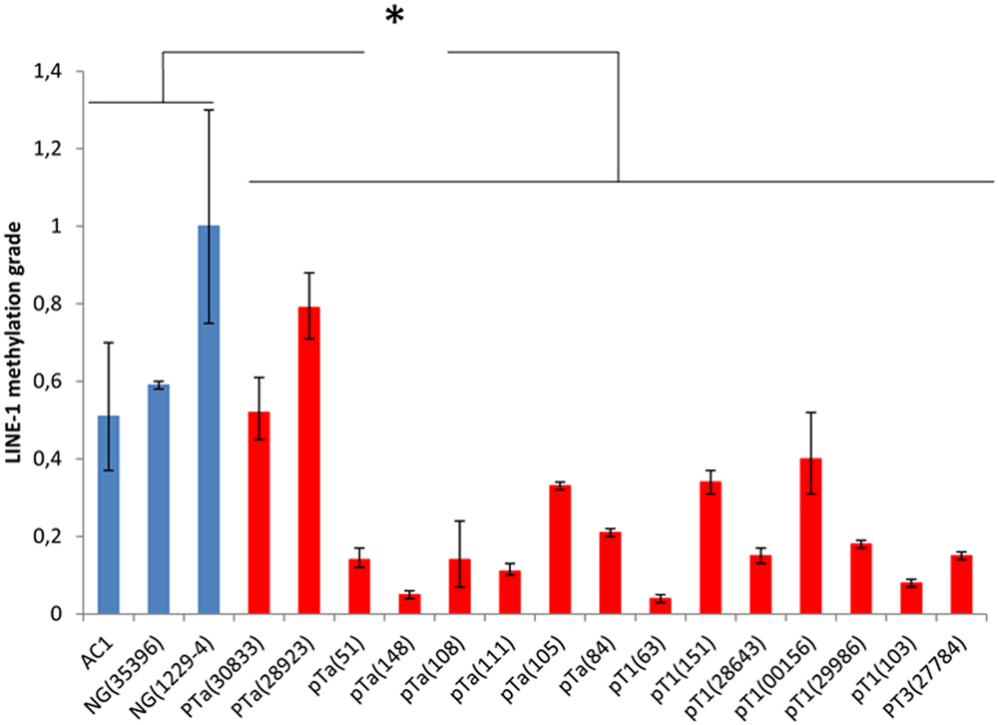
Relative quantification of LINE-1 methylation in UC. LINE-1 methylation was measured by real time IDLN-MSPCR in three healthy- and in eight pTa-, six pT1- and one pT3 UC samples, each with the corresponding sample ID (Table 1). Significant differences between the two groups (p < 0.05) were verified by the Student’s t-test*.

Global hypomethylation in the tissue specimens was measured by quantifying relative LINE-1 DNA methylation in the examined tissue samples by idiolocal normalized real-time methylation specific PCR^24^. This method allows a reliable comparison of LINE-1 methylation in tumor samples despite genetic heterogeneity and copy number changes^10^. Most UC tissues displayed marked hypomethylation in comparison to normal healthy urothelium (Fig. 6).

## Discussion

Urothelial carcinoma is broadly documented in the literature (reviewed by Schulz *et al*.)^25^ as a tumor entity with numerous and diverse epigenetic aberrations and associated genetic abnormalities that are thought to originate in the early stages of the carcinogenesis process and spreading across the entire genome. It should be emphasized that consequently the latter vastly interfere with and largely hamper reliable and comparative gene expression analyses in UC samples of different individuals, since it is technically not practicable to define beforehand any measurement, the actual copy numbers, neither of genetic loci of interest, nor, where methodically required, of endogenous references. For instance relative quantification of gene transcription using the common ΔΔct-Method which uses an endogenous reference gene will inevitably lead to a measured higher expression of the gene of interest if the reference locus is affected by loss of heterozygosity (LOH) and therefore by diminished transcription^24^.

Urothelial carcinoma is thought to be caused by chemical carcinogens. For instance, tobacco smoking, a main risk factor, is thought to exert its adverse effect by aromatic amines and other contained carcinogens, reaching urothelial cells via the urine, forming DNA adducts and causing mutations. The precise mechanisms are however not fully understood^14^. Even less understood is the origin of epigenetic aberrations, although some have recently been linked to chemical carcinogens (reviewed in Schulz & Goering)^8^.

In the present manuscript, we provide evidence that genes encoding key factors of methyl group metabolism could be among the first genes epigenetically altered during urothelial carcinogenesis. Specifically, using two independent techniques, we found increased DNA methylation within the promoter regions of the genes *ODC1*, *ACHY*, and *MTHFR* in early stage urothelial carcinoma tissues. As a consequence these genes may become epigenetically transcriptionally silenced or repressed. All three gene products influence the crucial cellular ratio of S-adenosyl-L-methionine (SAM) to S-adenosyl-L-homocysteine (SAH). SAM is the methyl group donor for many cellular methylation reactions, but especially for DNA methylation. SAH is generated from SAM in these reactions and efficiently inhibits SAM-dependent methyltransferases^26^. Accordingly, significantly decreased SAM levels or significantly increased SAH levels, i.e. any significant decrease of the SAM:SAH ratio leads to global DNA hypomethylation^26^. Global hypomethylation is a characteristic of many cancers, related to genomic instability^27,28^ and is particularly prominent in urothelial carcinoma^29^. Therefore, the hypermethylation of key methyl group metabolism genes in early urothelial carcinoma specimens may promote further epigenetic alterations such as global DNA hypomethylation. Beyond their possible involvement in the molecular etiology of the disease, DNA methylation changes in methyl group metabolism genes may be useful for diagnostic and prognostic purposes, monitoring of treatment and recurrence, and for the classification of urothelial carcinoma.

The first enzyme affected by these hypermethylation events, ODC1, ornithine decarboxylase, is the rate-limiting enzyme of polyamine biosynthesis. It thus belongs to the group of ‘housekeeping’ enzymes that are expressed in practically all tissues. Its 5′-regulatory region, which has been shown to be crucial for expression^30^, encompasses a CpG-island which should be always free of methylation.

In early work, a positive correlation between *ODC1* gene hypomethylation and expression was observed^31^, experimental methylation of *ODC1* abolished expression^32^ and aberrant methylation was reported in malignant cells^33^. Accordingly, we demonstrated here that the 5′-regulatory region of *ODC1* is hypermethylated in early UC (Fig. 2), has indeed promoter activity and that it can be efficiently repressed by DNA methylation (Supplementary Fig. 2a). ODC is tightly regulated at the protein level by multiple mechanisms^34^ that control its very rapid turnover, implying that transcriptional downregulation could lead to diminished enzyme activity within the cell. Interestingly, analysis of the pan-cancer TCGA data reveals that ODC1 expression in bladder cancer is among the lowest of any cancer type and substantially decreased compared to normal bladder cancer tissues (Suppl. Figure 3). ODC1 is linked to methyl group metabolism by the observation that interference with ODC1 enzyme activity results in accumulation of SAH and decarboxylated S-adenosylmethionine (dcSAM), which likewise acts as a competitive inhibitor of methylation reactions, resulting in genome-wide DNA demethylation, as shown e.g. in human oral cancer cells^35,36^. Thus, the dense DNA methylation encompassing the *ODC1* 5′-regulatory region in most of the analyzed early urothelial carcinoma specimens would be expected to epigenetically impair ODC1 expression.

Second, we have documented here DNA methylation of the 5′-regulatory region of *AHCY* encoding S-adenosylhomocysteine hydrolase in urothelial carcinoma specimens. Like *ODC1*, *AHCY* is transcribed from a single CpG-island promoter. In mammals, AHCY is the only known enzyme that catalysis the hydrolysis of SAH to homocysteine and adenosine, thereby relieving the inhibitory effect of SAH^37^. *AHCY* downregulation was previously shown to contribute to global DNA hypomethylation^38^ and to tumorigenesis, by conferring resistance to p53 and p16^INK4^-induced proliferation arrest^39^. Moreover, inhibition of S-adenosyl-*L*-homocysteine hydrolase and the resulting increase in SAH levels were shown to elicit DNA hypomethylation in cultured endothelial cells^40^. *AHCY* mRNA was found lost in 50% of tumor tissues from 206 patients with different kinds of tumors in comparison to their normal tissue counterparts; downregulation was found in 14 different tumor entities, including renal carcinoma, but unfortunately UC was not investigated in that study^39^. Taken together, these observations actually hint at a tumor suppressor function for AHCY, which is largely unexplored. Our observation of dense DNA methylation in the *AHCY* promoter suggests that also *AHCY* transcription too is epigenetically impaired in early UC. This could lead to SAH accumulation and DNA hypomethylation and might contribute to tumorigenesis.

Third, Methylenetetrahydrofolate reductase (MTHFR) catalyzes the conversion of 5,10-methylenetetrahydrofolate to 5-methyltetrahydrofolate, a co-substrate for remethylation of homocysteine to methionine. There is overwhelming evidence that reduced MTHFR expression in diverse tumor entities leads to homocysteine accumulation and DNA hypomethylation^41^. In various cases genetic polymorphisms, e.g. the two common functional polymorphisms A1298C (rs1801131) and C677T (rs1801133) which diminish enzymatic activity, are involved. For instance, carriers of the *MTHFR* variant allele A1289C had 4% lower LINE-1 methylation compared to those carrying the more common genotypes of this SNP in histologically normal breast tissues^42^. Interestingly, this allele was reported to confer a 4.76-fold increased risk of developing bladder cancer^43^. Decreased expression of *MTHFR* may also occur as a consequence of promoter hypermethylation. The 5′-region analyzed by us was identified as the site of focal epimutations in mothers of Down syndrome children associated with global LINE-1 hypomethylation in their blood cells^44^. Noteworthy, we demonstrate by a reporter assay that this region has promoter activity and is repressed by DNA methylation (Suppl. Figure 2c). In human lung cancer cells *MTHFR* methylation was inversely correlated with gene expression^45^. The increased methylation found in our study was also unusually caused by focal methylation of specific non-CpG-sites in the *MTHFR* 5′-regulatory region. Two of them form, according to *in silico* prediction, parts of GRα binding sites. GR expression tends to be weaker in bladder cancer tumors than in normal cells, and strong GR expression tends to be correlated with a better prognosis^46^. In addition, systemic use of glucocorticoids has been linked to an increased risk of bladder cancer^47^. Focal cytosine methylation at transcription factor binding sites may lead to altered gene expression^48^.

Taking together, we present evidence that in early UC altered DNA methylation occurs at genes encoding key factors of methyl group metabolism. Their altered expression is expected to interfere with methylome integrity and in a feed-forward loop may propagate the development of epigenetic abnormalities, e.g. LINE-1 hypomethylation and other epigenetic disturbances in early urothelial carcinoma. Indeed, we detected marked LINE-1 hypomethylation in UC samples which concomitantly exhibit DNA methylation of methyl group metabolic genes (Fig. 6).

On the basis of our study we suggest here the following hypothesis, named PrimeEpiHit (PEH) hypothesis as initial part of the molecular etiology of urothelial carcinoma.

Carcinogenic substances, e.g. aromatic amines from cigarette smoke exert their dangerous influence via the urine on urothelial cells. They are able to cross the cellular membranes, hit, react with and affect the genetic material. In particular they chemically interact with DNA to form mutagenic adducts and may interfere with proper transcription.

Due to the chronic character of this influence, occasionally also genes with key functions in methyl group metabolism are affected, by impairment of their transcription and subsequently altered epigenetic status. This impairment causes regulation imbalances within the involved methyl group metabolic pathways, disturbances of the delicate SAM:SAH ratio and consequently genome-wide DNA methylation alterations, including LINE-1 hypomethylation, ultimately resulting in genetic instabilities and cellular transformation. Noteworthy, this fatal development could be in parallel enhanced by the well described aging-dependent deficits of methyl group metabolism which are likewise characterized by an enhanced accumulation of SAH and DNA hypomethylation^49^. Age is also an important risk factor for bladder cancer^1^. Finally, it should be noted that such epigenetic alterations of genes with key importance in methyl group metabolism appear to fit well with the broadly discussed “few hits”-theory of cancer initiation. Here it has been suggested that for the most cancers few genetic, e.g. 6 for colon cancer, and in addition epigenetic events are together responsible for cancer initiation^50^.

## Methods

### Preparation *of* DNA from formalin-fixed, paraffin-embedded (**FFPE**) tissue samples and punches

**Figure 7:**
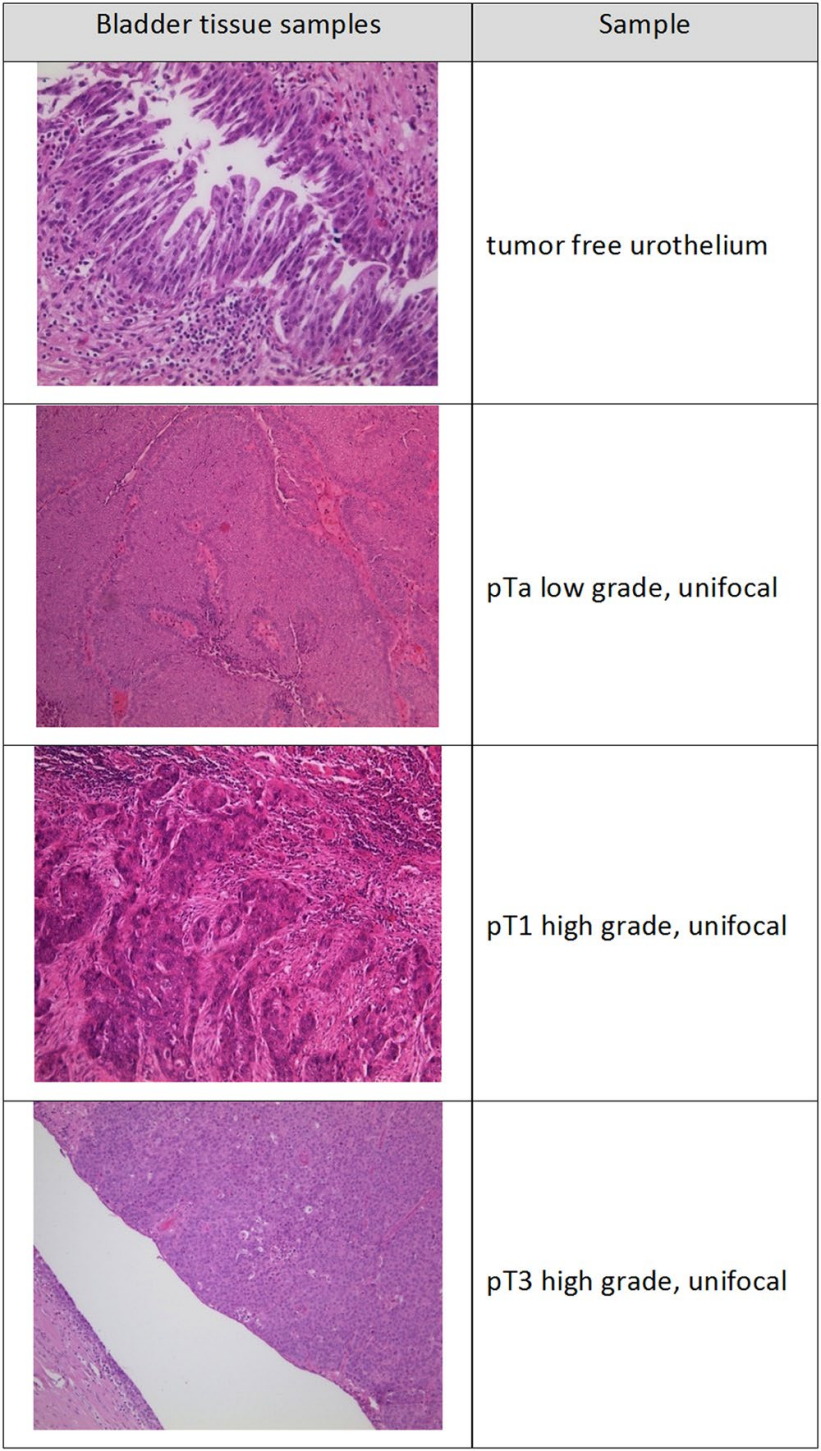
Representative pictures of pathologically classified tissue samples used for methylation analysis.

agenode) according to the All methods were carried out in accordance with relevant guidelines and regulations. We confirm that the experimental protocols were approved and informed consent was obtained from all participants. An appropriate ethics vote was granted by the Kantonale Ethikkommission Zürich on 22.02.2013, Ref. Nr. KEK-ZH-Nr 2012–0352. Histological H&E stained sections from formalin-fixed paraffin-embedded (FFPE) tissue specimens (UC and non-dysplastic urothelium of individuals without a history of UC) were reviewed for tumor and urothelium content and the target area was marked by a trained pathologist (Dr. Jankowiak, Dr. Braunstein, Dr. Anlauf, Dr. Wild or Dr. Buser) for tissue punching using a hollow needle. DNA from FFPE punches (3 cylinders with diameter of 0.6 mm and a length of 2–3 mm) was isolated using the Maxwell 16 FFPE Tissue LEV DNA Purification Kit (Promega, Madison, United States, #AS1130) according to the manufacturer’s recommendations. Briefly, FFPE cylinders were deparaffinised with xylene, washed twice with ethanol, dried 10 min at 37 °C and resuspended in 200 μl incubation buffer containing 2 mg/ml proteinase K. Samples were incubated overnight at 70 °C and mixed with 400 μl lysis buffer. Lysates from FFPE tissue were transferred to well 1 of the supplied cartridge of the corresponding kit and DNA was automatically purified and eluted in 30 μl Tris-buffer, pH 8.0 by the Maxwell instrument. The yield ranged between 1 and 8 µg of high quality DNA per sample. Purity control and quantification were performed using a NANODROP 2000 UV-Vis spectrometer (Thermo SCIENTIFIC, Wilmington, USA). Table 1 and Fig. 7 provide an overview of all used clinical samples and representative histological images (Table 1, Fig. 7). Overall we obtained five punches from multifocal UC tissue and five punches from the respective adjacent, benign urothelial tissue. These 5 UC punches consisted of 2pTa low grade, 1pTa high grade and 2 pT1 high grade tumors. Furthermore we obtained one microdissected, unifocal, pTa low grade UC tissue sample, eight punches of early unifocal UC and four punches with respective adjacent, benign urothelial tissue. In addition, we obtained one microdissected pT3 high grade unifocal UC tissue sample. As references we obtained two paraffin-embedded, microdissected tissue samples and two punches of healthy urothelium. The eight punched samples of early unifocal UC were three pTa low grade, two pTa high grade, one pT1 low grade and three pT1 high grade tumors. Each of the few samples for microdissection was pathologically reviewed and the marked region of interest was cut into 5 µm slices which were transferred into Eppendorf reaction tubes. Tissue was dewaxed by incubation in xylene 2 times for 10 minutes followed by ethanol series 100% to 60% for 5 min each. Samples were cleaned in bidest. H_2_O and dried at RT. Cellular DNA was isolated using the OIAamp DNA Mini Kit (Qiagen, Hilden, Germany) according to the manufacturer’s protocols.

### Methylated DNA Immunoprecipitation (MeDIP)

Genomic DNA (1 µg) dissolved in a final volume of 100 µl was sonicated at 4 °C in TPX® polymethylpentene tubes using a Bioruptor® sonicator (Diagenode, Liege, Belgium) in order to produce random sized fragments ranging from 300–1.000 bp. Twenty sonication pulses of each 15 sec were applied. Immunoprecipitation (IP) of methylated DNA was then performed by magMeDIP kit (Diagenode) according to the manufacturer’s instructions. Briefly, DNA samples at a final concentration of 0.1 µg/µl were denatured at 95 °C for 3 min., quickly chilled on ice and immunoprecipitated by adding 20 µl magnetic bead-immobilized 5-methylcytosine (5-mC) monoclonal 33D3 antibody (Diagenode). The bound DNA was separated from the beads by incubation with proteinase K at 55 °C for 15 min. Control input samples contained 20% of the corresponding DNA sample used for IP.

### Amplification and labelling of DNA

Whole genome amplification of the input DNA and the immunoprecipitated DNA samples was performed applying the Genome Plex Complete WGA Kit (Sigma-Aldrich, St. Louis, United States) as described by the user’s guide. The amplification products were purified using QIAquick PCR Purification Kit (Qiagen, Hilden, Germany). Input and IP DNA were labeled with Cyanine 3 and Cyanine 5, respectively, using random primers and Klenow fragment polymerase. Labeling was performed using the SureTag Complete DNA Labeling Kit (Santa Clara, CA), as suggested by the manufacturer. Labeled DNA was cleaned with 70% ethanol and dried using a vacuum centrifuge for few minutes.

### Hybridization of microarrays

Equal amounts of labeled IP and input DNA (700 ng) were combined and loaded on NimbleGen 385 K RefSeq Promoter Arrays HG18, containing all known human RefSeq genes (Roche, Basel, Switzerland) or NimbleGen Human DNA Methylation 2.1 M Deluxe Promoter Arrays. Initially, tissue samples from Düsseldorf were analyzed by NimbleGen 385 K Arrays; after the upgraded, high resolution 2.1 M NimbleGen Array became available, tissue samples from Zürich were analyzed on these arrays.

On both arrays all known gene 5′-regulatory regions are covered by 75-mer probes with approximately 100-bp spacing. The 385 K arrays cover a region between 2.2 kb downstream and 0.5 kb upstream of the transcription start site. The 2.1 M arrays cover a region between 7.25 kb downstream and 3.25 bp upstream of the transcription start site. The hybridization procedure was executed at 42 °C for 16 h in the NimbleGen hybridization chamber in accordance with the manufacturer’s protocol. The hybridized arrays were washed thoroughly and dried using a microarray centrifuge for 1 min in the dark.

### DNA methylation microarray scanning and data analyses

The hybridized arrays were scanned on an MS200 microarray scanner (Roche, Basel, Switzerland) at a resolution of 2 µm. The raw methylation data were extracted with the default ChIP protocol from software NimbleScan for 385 K arrays and with DEVA for 2.1 M arrays. Methylation ratios between the IP DNA samples and the control input samples were normalized across samples using the quantile method after performing a variance stabilization using log2 scaling for each promoter feature on the array. All data processing including mapping of microarray probes to promoters, gene annotation, data post-processing, principal component analysis, identification of differentially methylated regions, and graphics were performed with in-house developed functions in Matlab^51,52^.

### Determination of differentially methylated regions (DMRs) of gene promoters

The promoter loci information on the DNA methylation microarray probes was taken from NimbleGen annotation information based on RefSeq version MM9. For each selected pair of sample subgroups, we calculated the mean of each probe across all samples of the subgroup. To address the statistical significance of the differently methylated regions (DMRs) we applied the two-samples Student’s t-test with a significance threshold αDMR = 0.05. The level of significance was set to p < 0.05. This means that a gene with such a value has a probability of >95% to show significant hypermethylation within the appropriate group by direct comparison to the reference group.

### Bisulfite genomic sequencing

Bisulfite sequencing was performed following bisulfite conversion with the EpiTec Kit (Qiagen, Hilden, Germany) as described (Santourlidis *et al*. 2002, Ghanjati *et al*.)^10^. Primers were designed after excluding pseudogenes or other closely related genomic sequences which could interfere with specific amplification by amplicon and primer sequences comparison in BLAT sequence data base (https://genome.ucsc.edu/FAQ/FAQblat.html). The following PCR primers for amplification of *AHCY*, *CBS*, *MTHFR* and *ODC1* 5′-regulatory gene regions by nested PCR have been used (Table 2).

**Table 2:** Primers used for bisulfite genomic sequencing.

Primer Name	Sequence	Product Length
s1AHCYkonv	5′–ATTTTGAGGTTTTTTTTTAGGGA–3′	348 bp
as1AHCYkonv	5′–TTCCAAAAAATCCAAAAAACC–3′	
as2AHCYkonv	5′–CAAAAAATCCAAAAAACCCCC–3′	
s1CBSkonv	5′–GTGATTTTAAGGGGGTTTTG–3′	362 bp
s2CBSkonv	5′–ATTTTAAGGGGGTTTTGTG–3′	
as1CBSkonv	5′–TCTAACACAAACCCTATCTAACC–3′	
as2CBSkonv	5′–CACAAACCCTATCTAACCTAAAA–3′	
s1MTHFRkonv	5′–TAGGAGGGGTTATGAGAAAAGAT–3′	450 bp
s2MTHFRkonv	5′–GAGGGGTTATGAGAAAAGATTTT–3′	
as1MTHFRkonv	5′–TCCTAATCTCAATCCCAAAACTC–3′	
as2MTHFRkonv	5′–ACTCCTAATCTCAATCCCAAAAC–3′	
s1ODC1konv	5′–GGTTTTGTTAGTTTTTTTTGTA–3′	224 bp
s2ODC1konv	5′–TTTTGTTAGTTTTTTTTGTAGT–3′	
as1ODC1konv	5′–AAAAATCCCTCACCTCAA–3′	
as2ODC1konv	5′–AAATGCCTCACCTCAAAA–3′	

In brief, the pre-amplification conditions were denaturation at 95 °C for 13 min. followed by 22 cycles of 95 °C for 50 s, TM for 45 s (AHCY: 56 °C; CBS: 54 °C; MTHFR: 51 °C; ODC1: 52 °C), and 72 °C for 30 s. For the second round of the applied nested PCR the pre-amplification product was diluted 1:10 and the same PCR conditions were chosen for 35 additional cycles with the second primer pair. The amplification products were 348 bp (AHCY), 362 bp (CBS), 450 bp (MTHFR) and 224 bp (ODC1) in size. Amplification products were cloned into pCR2.1 vector using the TA Cloning Kit (Invitrogen, Carlsbad, United States) according to the manufacturer’s instructions. On average 30 clones were sequenced using the BigDye Terminator Cycle Sequencing Kit (Applied Biosystems, Foster City, United States) on a DNA analyzer 3700 (Applied Biosystems) with M13 primer to obtain a representative methylation profile of each sample. 5′-regulatory gene sequences refer to +1 transcription start of the following sequences:

Homo sapiens ornithine decarboxylase 1 (ODC1), transcript variant 1, mRNA

NCBI Reference Sequence: NM_002539.2

Homo sapiens adenosylhomocysteinase (AHCY), transcript variant 1, mRNA

NCBI Reference Sequence: NM_000687.3

Homo sapiens methylenetetrahydrofolate reductase (NAD(P)H) (MTHFR), mRNA

NCBI Reference Sequence: NM_005957.4

Homo sapiens cystathionine-beta-synthase (CBS), transcript variant 1, mRNA

NCBI Reference Sequence: NM_000071.2

### Relative Quantification of LINE-1 methylation in UC and reference samples by real-time MSP

Real time Methylation-Specific PCR of differentially methylated LINE-1 promoter regions was performed as following: Converted DNA of the used tissue samples served as template for amplification of methylated LINE-1 sequences in a normalized real time MSP approach for genetic imbalanced DNA specimens as described (Santourlidis *et al*.)^24^. The primers listed below have been used (Table 3).

**Table 3:** Primers used for LINE-1 real time Methylation-Specific PCR.

Primer Name	Sequence	Product Length
sL1met	5′-GCGCGAGTCGAAGTAGGGC-3′	193
asL1met	5′-CTCCGACCAAATATAAAATATAATCTCG-3′	
sL1control	5′-AGGTTTTATTTTTGGGGGTAGGGTATAG-3′	207
asL1control	5′-CCCCTACTAAAAAATACCTCCCAATTAAAC-3′	

The amplification conditions were denaturation at 95 °C for 10 min, followed by 40 cycles of 95 °C for 30 s, TMs: 61 °C for 40 s (L1met) or 58 °C for 40 s (L1control) and 72 °C for 15 s.

### Luciferase reporter assay and cell lines

The human epithelial, embryonal kidney cell line HEK 293 T, established from a human primary embryonal kidney transformed by adenovirus type 5 was cultured according to the manufacturer’s recommendation in high glucose DMEM GLUTAMAX (Gibco, Waltham, United States) containing 10% FCS (Gibco) and 1% penicillin and streptomycin at 37 °C and with 5% CO2.

The gene promoter fragments were amplified using the primers listed below and cloned into the pGL3 basic vector using the restriction enzymes XhoI and HindIII (NEB, Ipswich, United States). All constructs were validated by sequencing. For analysis of the influence of promoter methylation the constructs were *in vitro* methylated by using the CpG methyltransferase M. SssI (NEB).

For the Luciferase Assay the cells were seeded into 48-well plates. The cells were cotransfected with each 50 ng of the pGL3 basic vector (Promega, Madison, United States) and pTK-Green Renilla vector (Invitrogen, Carlsbad, United States). Lipofectamine 2000 (Invitrogen) was used as transfection reagent according to the manufacturer’s suggestion. The following primers have been used (Table 4).

**Table 4:** Primers used for luciferase reporter assay.

Primer Name	Sequence	Product Length/TM
s1ODC1	5′-CCGCTCGAGCGGATAAGTAGGGAGCGGCGTG-3′	298 bp/58 °C
as1ODC1	5′-CCCAAGCTTGGGCTCCCTCCCTTCCTCCG-3′	
s1AHCYRS	5′-CCGCTCGAGCGGAGTTCCGCTGGGTTTTGAC-3′	503 bp/60 °C
as1AHCYRS	5′-CCCAAGCTTGGGATTCCAGGGGGTCCAGAGA-3′	
s2MTHFRRS	5′-CCGCTCGAGCGGCCAGGAGGGGTTATGAGAAAAGAC-3′	456 bp/62 °C
as2MTHFRRS	5′-CCCAAGCTTGGGCTCGCCCCACCCGTCTG-3′	

The Luciferase Assay was performed with the Dual Luciferase Reporter Assay system (Promega) according to the manufacturer’s guideline. In short, growth medium was removed and the cells were washed with PBS (Gibco). To each well 65 µl of passive lysis buffer was dispensed and the cells were incubated for 15 minutes at room temperature on a rocking platform. Twenty µl of cell lysate were then transferred into each luminometer tube and 100 µl Luciferase Assay Reagent II was added. Firefly luciferase activity was measured by a 2-second premeasurement delay, followed by a 10-second measurement period for each reporter assay with the Lumat LB 9507 (Berthold Technologies, Bad Wildbad, Germany). The firefly luciferase reaction was stopped by adding 100 µl Stop & Glo reagent and Renilla luciferase activity was measured.

## Electronic supplementary material

Dataset 1
