# Author Correction: Aberrant methylated key genes of methyl group metabolism within the molecular etiology of urothelial carcinogenesis

**DOI:** 10.1038/s41598-018-23158-z

**Published:** 2018-04-11

**Authors:** Lars Erichsen, Foued Ghanjati, Agnes Beermann, Cedric Poyet, Thomas Hermanns, Wolfgang A. Schulz, Hans-Helge Seifert, Peter J. Wild, Lorenz Buser, Alexander Kröning, Stefan Braunstein, Martin Anlauf, Silvia Jankowiak, Mohamed Hassan, Marcelo L. Bendhack, Marcos J. Araúzo-Bravo, Simeon Santourlidis

**Affiliations:** 1 Epigenetics Core Laboratory, Institute of Transplantation Diagnostics and Cell Therapeutics, Medical Faculty, Heinrich-Heine University Duesseldorf, Moorenstr. 5, 40225 Duesseldorf, Germany; 2 Department of Urology, University Hospital, University of Zurich, Zurich, Switzerland; 3 Department of Urology, Medical Faculty, Heinrich-Heine University Duesseldorf, Duesseldorf, Germany; 4 Urologische Klinik, Universitätsspital Basel, Basel, Switzerland; 5 Institute of Surgical Pathology, University Hospital, University of Zurich, 8091 Zurich, Switzerland; 6 Department of Pathology, Medical Faculty, Heinrich-Heine University Duesseldorf, Duesseldorf, Germany; 7 Department of Surgery, Tulane University School of Medicine, New Orleans, LA 70112 USA; 8 Institut National de la Santé et de la Recherché Médicale, University of Strasbourg, 67000 Strasbourg, France; 9 Department of Urology, University Hospital, Positivo University, Curitiba, Brazil; 10 Group of Computational Biology and Systems Biomedicine, Biodonostia Health Research Institute, 20014 San Sebastián, Spain; 11 IKERBASQUE, Basque Foundation for Science, 48009 Bilbao, Spain

Correction to: *Scientific Reports* 10.1038/s41598-018-21932-7, published online 22 February 2018

The original version of this Article contained a typographical error in the spelling of the author Marcos J. Araúzo-Bravo, which was incorrectly given as Marcos-Arauzo Bravo. This has now been corrected in the PDF and HTML versions of the Article, and in the accompanying Supplementary Information file.

