# Supplementary material for: Aberrant methylated key genes of methyl group metabolism within the molecular etiology of urothelial carcinogenesis
Source: Sci Rep. 2018 Feb 22;8:3477. doi: 10.1038/s41598-018-21932-7 (PMC5823913; doi:10.1038/s41598-018-21932-7)
Supplement: Supplementary file 1 — Dataset 1 [file 41598_2018_21932_MOESM1_ESM.docx]

**Aberrant methylated key genes of methyl group metabolism within the molecular etiology of urothelial carcinogenesis**

**Lars Erichsen^1^, Foued Ghanjati^1^, Agnes Beermann^1^, Cedric Poyet^2^, Thomas Hermanns^2^, Wolfgang A. Schulz^3^, Hans-Helge Seifert^4^, Peter J. Wild^5^, Lorenz Buser^5^, Alexander Kröning^1^, Stefan Braunstein^6^, Martin Anlauf^6^, Silvia Jankowiak^6^, Mohamed Hassan^7^, Marcelo L. Bendhack^8^, Marcos J. Araúzo-Bravo^9^, Simeon Santourlidis^1^***

^1^Epigenetics Core Laboratory, Institute of Transplantation Diagnostics and Cell Therapeutics, Medical Faculty, Heinrich-Heine University Duesseldorf, Moorenstr. 5, 40225 Duesseldorf, Germany.

^2^Department of Urology, University Hospital, University of Zurich, Zurich, Switzerland.

^3^Department of Urology, Medical Faculty, Heinrich-Heine University Duesseldorf, Germany

^4^Urologische Klinik, Universitätsspital Basel.

^5^Institute of Surgical Pathology, University Hospital, University of Zurich, 8091 Zurich, Switzerland

^6^Department of Pathology, Medical Faculty, Heinrich-Heine University Duesseldorf, Germany.

^7^Department of Surgery, Tulane University School of Medicine, New Orleans, LA 70112, USA and Institut National de la Santé et de la Recherché Médicale, U1121, 75019 Paris, France

^8^Department of Urology, University Hospital, Positivo University, Curitiba, Brazil

^9^Group of Computational Biology and Systems Biomedicine, Biodonostia Health Research Institute, 20014 San Sebastián, Spain; IKERBASQUE, Basque Foundation for Science, 48009 Bilbao, Spain.

*Correspondence and requests for materials should be addressed to S.S.

(email: [simeon.santourlidis@med.uni-duesseldorf.de](mailto:simeon.santourlidis@med.uni-duesseldorf.de))

Urothelial carcinoma (UC), the most common cancer of the urinary bladder causes severe morbidity and mortality, e.g. about 40.000 deaths in the EU annually, and incurs considerable costs for the health system due to the need for prolonged treatments and long-term monitoring. Extensive aberrant DNA methylation is described to prevail in urothelial carcinoma and is thought to contribute to genetic instability, altered gene expression and tumor progression. However, it is unknown how this epigenetic alteration arises during carcinogenesis. Intact methyl group metabolism is required to ensure maintenance of cell-type specific methylomes and thereby genetic integrity and proper cellular function. Here, using two independent techniques for detecting DNA methylation, we observed DNA hypermethylation of the 5´-regulatory regions of the key methyl group metabolism genes *ODC1*, *AHCY* and *MTHFR* in early urothelial carcinoma. These hypermethylation events are associated with genome-wide DNA hypomethylation which is commonly associated with genetic instability. We therefore infer that hypermethylation of methyl group metabolism genes acts in a feed-forward cycle to promote additional DNA methylation changes and suggest a new hypothesis on the molecular etiology of urothelial carcinoma.

Supplementary data

**Suppl. fig. 1: Mean Peak Values detected by microarray analysis for the genes *AHCY, AHCYL2, MTHFR* and *ODC1* in each respective subgroup.** The mean values are for *AHCY*: Control: 22085, UT: 33280, bG UT: 28438, MT: 27775, bG MT: 27500 *AHCYL2*: Control: 22971, U: 29357, bG UT: 32778, MT: 32726, bG MT: 31793 *MTHFR*: Control: 20893, UT: 27891, bG UT: 32515, MT: 29866, bG MT: 30509 *ODC1*: Control: 24090, UT: 32152, bG UT: 31552, MT: 32356, bG MT: 30719. Abbreviation “bG” stands for benign, tumor adjacent tissue sample.

**Suppl. fig. 2a: Repression of luciferase reporter by DNA methylation of 5´-regulatory gene region of *ODC1* gene in HEK 293 T cells**

The epithelial embryonic kidney cell line HEK 293T was transfected with 50ng of each of the following constructs: pGL3 basic vector, pGL3 + *ODC1* 5´-regulatory region unmethylated and both *in vitro* methylated by M. SssI CpG methyltransferase. Relative Firefly luciferase activity was determined and is given in percent.

Transfection of HEK-293 T cells with miR-133b significantly suppressed a luciferase-reporter containing the Bcl-w or Akt 1 3'-untranslated regions.

**Suppl. fig. 2b: Repression of luciferase reporter by DNA methylation of 5´-regulatory gene region of *AHCY* gene in HEK 293 T cells**

The epithelial embryonic kidney cell line HEK 293T was transfected with 50ng of each of the following plasmid constructs: pGL3 basic vector, pGL3 + *AHCY* 5´-regulatory region unmethylated and both *in vitro* methylated by M. SssI CpG methyltransferase. Relative Firefly luciferase activity was determined and is given in percent.

Transfection of HEK-293 T cells with miR-133b significantly suppressed a luciferase-reporter containing the Bcl-w or Akt 1 3'-untranslated regions.

**Suppl. fig. 2c: Repression of luciferase reporter by DNA methylation of 5´-regulatory gene region of *MTHFR* gene in HEK 293 T cells**

The epithelial embryonic kidney cell line HEK 293T was transfected with 50ng of each of the following constructs: pGL3 basic vector, pGL3 + *MTHFR* 5´-regulatory region unmethylated and both *in vitro* methylated by M. SssI CpG methyltransferase. Relative Firefly luciferase activity was determined and is given in percent.

Transfection of HEK-293 T cells with miR-133b significantly suppressed a luciferase-reporter containing the Bcl-w or Akt 1 3'-untranslated regions.


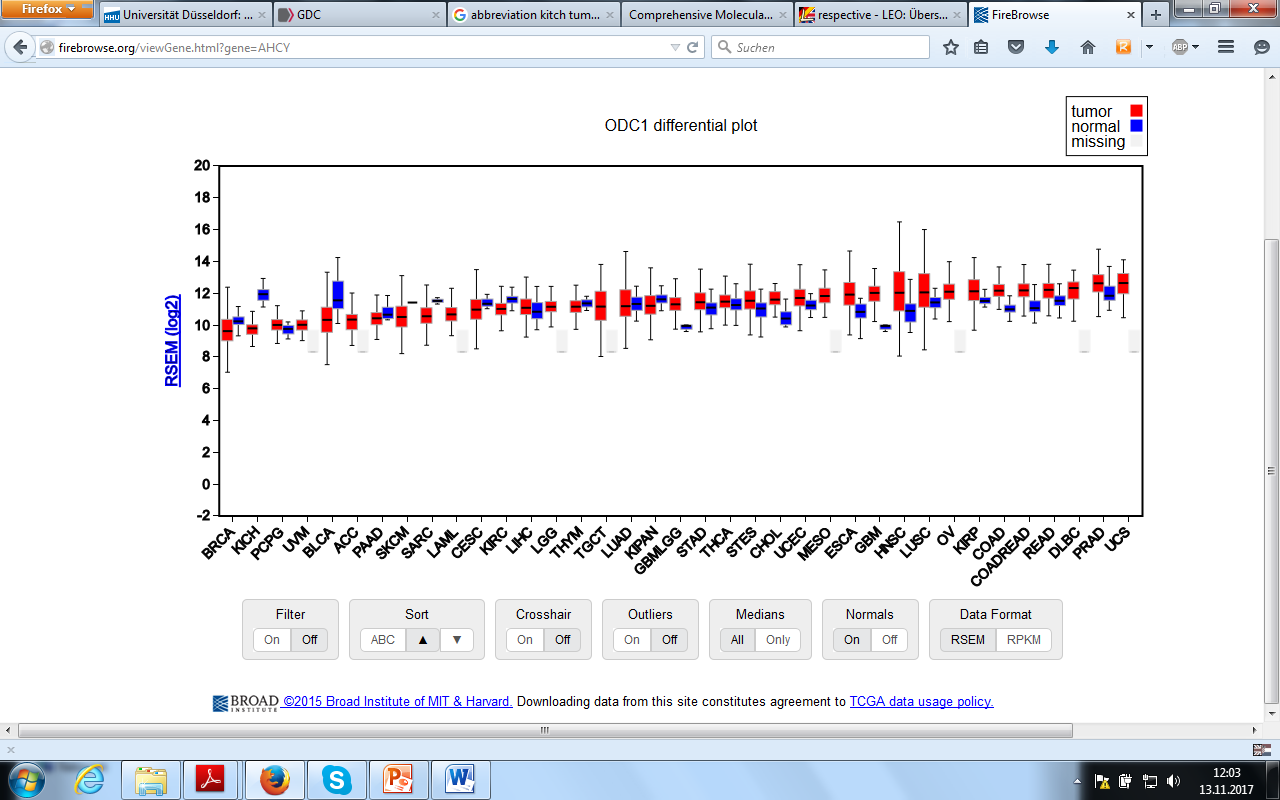


A


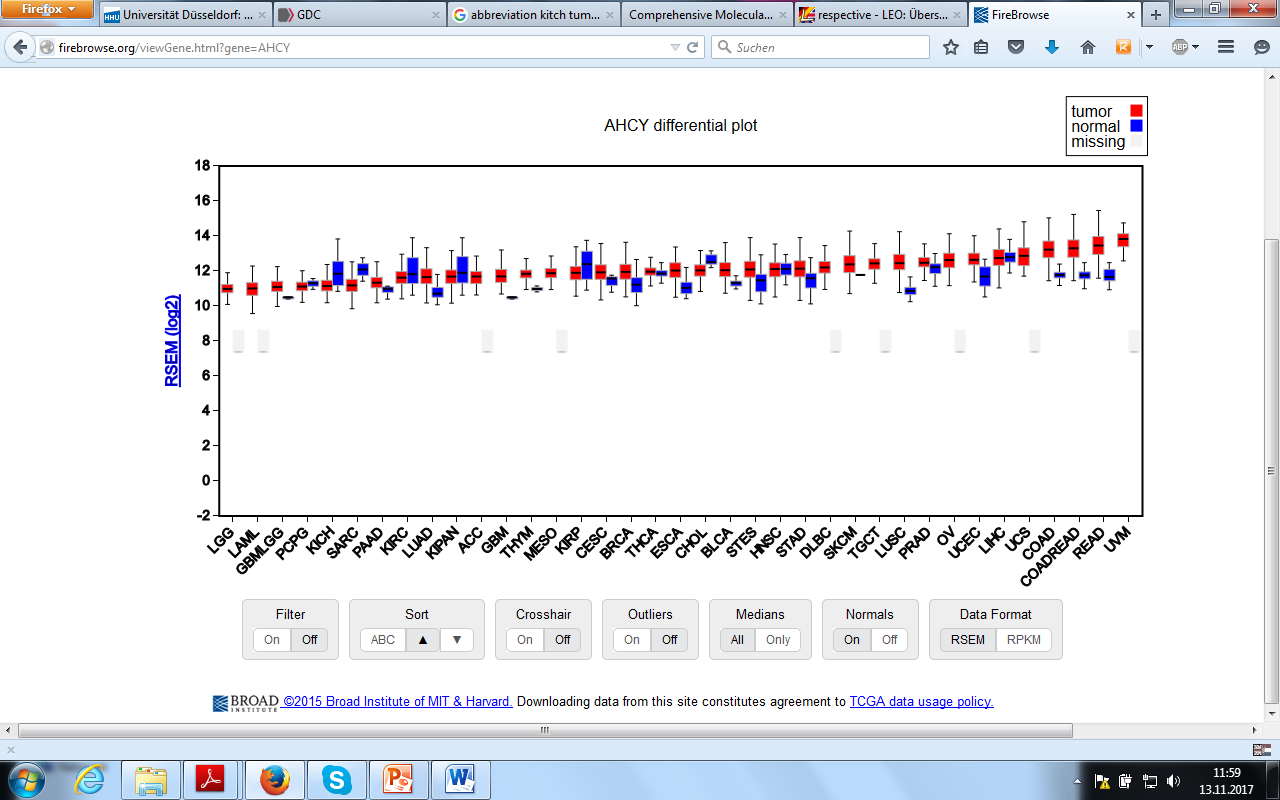


B


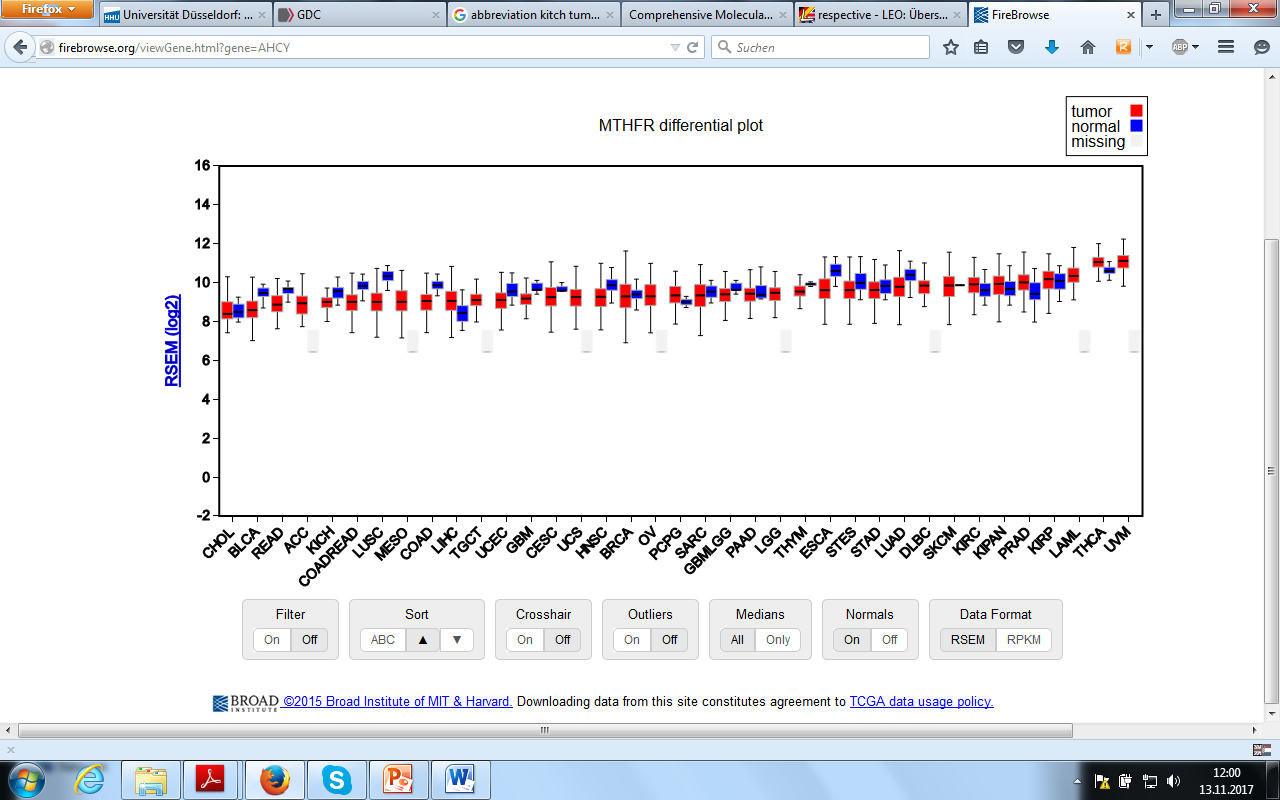


C

**Suppl. fig. 3: *ODC1*, *AHCY* and *MTHFR* mRNA expression in diverse tumor entities.**

The figure shows *ODC1* (A), *AHCY* (B) and *MTHFR* (C) mRNA expression in diverse tumor tissues (red) in comparison to the respective healthy tissues (blue) quantified by RNA-seq (TCGA), analyzed by RNA sequencing Expectation-Maximization as described by Li and Dewey (2011) and plotted as log_2_ RSEM. The mean score for each cohort is indicated by a horizontal black bar. Missing data are indicated by white boxes. Tumor types are arranged from left to the right by increasing expression. For instance, bladder cancer samples show the fifth lowest *ODC1* expression and uterine carcinosarcoma shows the highest *ODC1* expression. For bladder cancer, *ODC1*, *AHCY* and *MTHFR* expression in 408 cancer samples is compared to 19 samples of healthy urothelial tissue. They were 408 chemotherapy-naive, invasive, high-grade urothelial tumors (T1 [n = 1], T2–T4a, N0–3, M0–1) (Robertson et al., 2017). Note lower expression of *ODC1* and *MTHFR* but not of *AHCY* in bladder tumors compared to healthy urothelial tissue.

| Sample ID | 30833 | 28923 | 51 | 148 | 108 | 111 | 105 | 84 | 63 | 151 | 28643 | 00156 | 29986 | 103 | 27784 |
| --- | --- | --- | --- | --- | --- | --- | --- | --- | --- | --- | --- | --- | --- | --- | --- |
| *ODC1* | ND | ND | + | = | + | + | + | + | + | + | ND | ND | + | + | ND |
| *AHCY* | ND | + | + | + | + | + | + | + | + | ND | + | ND | ND | ND | ND |
| *MTHFR* | ND | ND | + | = | = | = | ND | ND | + | + | ND | ND | ND | ND | ND |
| *LINE 1* | 0,52 | 0,79 | 0,14 | 0,05 | 0,14 | 0,11 | 0,33 | 0,21 | 0,04 | 0,34 | 0,15 | 0,4 | 0,18 | 0,08 | 0,15 |

**Suppl. table. 1: *ODC1*, *AHCY* and *MTHFR* methylation status of the samples (see table 1) of which the LINE-1 methylation status is presented in figure 6**

The methylation status of the three genes has been determined by DNA methylation array technology. + means hypermethylated. = means hypomethylated as in the controls. ND means not determined. The last line depicts the corresponding absolute values of LINE-1 methylation as graphically illustrated in figure 6.
